# The Relationship between Cerebral White Matter Integrity and Cognitive Function in Mild Stroke with Basal Ganglia Region Infarcts

**DOI:** 10.1038/s41598-018-26316-5

**Published:** 2018-05-30

**Authors:** Li-Jun Zuo, Zi-Xiao Li, Rong-Yan Zhu, Yao-Jing Chen, YanHong Dong, Yi-Long Wang, Xing-Quan Zhao, Zhan-Jun Zhang, Perminder Sachdev, Wei Zhang, Yong-Jun Wang

**Affiliations:** 1 Department of Neurology, Beijing Tiantan Hospital, Capital Medical University, Beijing, 100050 China; 2 China National Clinical Research Center for Neurological Diseases, Beijing, 100050 China; 3 State Key Laboratory of Cognitive Neuroscience and Learning, Beijing Normal University, Beijing, P.R. China; 4 Department of Pharmacology, Yong Loo Lin School of Medicine, National University Health System, Singapore, Singapore; 5 Centre for Healthy Brain Ageing (CHeBA), School of Psychiatry, UNSW Medicine, University of New South Wales, Sydney, NSW 2052 Australia; 6 Tiantan Clinical Trial and Research Center for Stroke, Beijing Tiantan Hospital, Capital Medical University, Beijing, 100050 China; 7 Vascular Neurology, Beijing Tiantan Hospital, Capital Medical University, Beijing, 100050 China; 8 Neuropsychiatric Institute, Prince of Wales Hospital, Randwick, NSW 2031 Australia; 9 Department of Geriatrics, Beijing Tiantan Hospital, Capital Medical University, Beijing, 100050 China; 10 Key Laboratory for Neurodegenerative Disorders of the Ministry of Education, Capital Medical University, Beijing, 100069 China; 11 Beijing Institute for Brain Disorders, Capital Medical University, Beijing, 100069 China; 12 Beijing Key Laboratory on Parkinson disease, Beijing, 100053 China

## Abstract

Mild stroke is a known risk factor for dementia. The relationship between cerebral white matter (WM) integrity and cognitive impairment (CI) in mild stroke patients with basal ganglia region infarcts is unknown. Total of 33 stroke patients and 19 age-matched controls underwent diffusion tensor imaging scans and a formal neuropsychological test battery. CI was defined as having a performance score 1.5 SD below the established norm. We compared the differences in Z-scores and Fraction Anisotropy (FA) values among controls, stroke with no CI (NCI) and stroke with CI groups. Multiple linear regressions were performed between FA values in affected regions and neuropsychological tests in stroke patients. The majority of stroke patients were in their 50s (56.90 ± 9.23 years). CI patients exhibited a significantly decreased Z score in visual delayed memory and remarkably decreased FA values in the right external capsule and right fornix (FWE-corrected) compared with NCI patients and controls. In stroke patients, the FA value in the right fornix was positively correlated with delayed visual memory. Mild stroke with basal ganglia region infarcts may be related to widespread abnormality of WM integrity. The lower WM integrity in the right fornix may be a marker of impaired delayed visual memory.

## Introduction

Patients with mild stroke [median National Institute of Health Stroke Scale (NIHSS) = 2, median modified Rankin Score (mRS) = 2] exhibit mild neurological symptoms^1^, but often suffer from cognitive impairment (CI). Our previous study found that the prevalence of cognitive impairment in a Chinese sample of mild stroke and transient ischemic attack (TIA) reached up to 59%^2^. CI is commonly reported in stroke patients with basal ganglia region infarcts^3,4^. The basal ganglia regions are a group of nuclei at the base of the forebrain that are strongly connected to the cortex. While the role of the basal ganglia regions have historically been restricted to motor function, recent research suggested that the basal ganglia regions were also involved in a variety of cognitive functions^5^. The basal ganglia regions contained the corpus striatum, claustrum, amygdaloid nucleus, subthalamic nucleus and internal capsule. The corpus striatum comprised the caudate nucleus and the lenticular nucleus; the lenticular nucleus was a collective name given to the putamen and globus pallidus. Basal ganglia served as a fundamental building block of performing cognitive tasks of various complexity^6–8^. Numerous studies have demonstrated that ischemic lesions in basal ganglia regions were related to the impairment of memory, learning, visuospatial skills, and attention^4,9,10^ and this cognitive impairment was mostly ascribed to the disruption of cereberollo-basal ganglia thalamo-cortical loops^5,11^. Recent study showed that an ischemic lesion after right middle cerebral artery stroke could affect remote white matter integrity, which was associated with poorer cognitive recovery^12,13^. It is unknown whether patients with acute basal ganglia region infarcts experienced abnormality of white matter integrity in remote areas, and whether this was related to cognitive dysfunction.

Diffusion tensor imaging (DTI), a MRI based technique, has proved to be a valuable tool in detecting, visualizing and quantifying the integrity of the cerebral microstructure by measuring the diffusion of water in tissue^14^. Fractional anisotropy (FA) is an index to identify white matter lesions and evaluate the integrity of white matter tracts^15,16^. There is increasing evidence showing that, apart from remote neurophysiological changes, such as decrease of cerebral blood flow, disruption of energy metabolism or Wallerian degeneration, an ischemic lesion could affect white matter integrity after stroke^17–19^, which has been associated with a worse memory performance^20,21^. A few small neuroimaging studies in older patients with stroke found that ischemic lesions could cause loss of white matter integrity in the ipsilesional and contralesional hemispheres^22–24^ and this was associated with poor cognitive function after stroke^13^. It is unknown whether lower microstructural integrity in focal regions or areas remote from stroke with basal ganglia region infarcts, including as remote as the contralesional hemisphere, was related to cognitive dysfunction after stroke. This has never been investigated in mild stroke with basal ganglia region infarcts. The mechanisms of cognitive impairment after stroke with basal ganglia region infarcts is especially important in mild stroke survivors, as they have mild neurological deficits but are often in a demanding phase of their lives with respect to working and family-related functioning.

We hypothesized that cognitive impairment after stroke with basal ganglia region infarcts would show a lower white matter structural integrity remote from the initial stroke and that white matter integrity might predict cognitive impairment after stroke.

## Methods

### Stroke patients

Total 33 first-ever mild stroke patients with basal ganglia region infarcts were recruited, with no other cortical or subcortical cerebrovascular abnormality on MRI, as well as 19 healthy controls matched for age, sex, education and handedness. Patients were recruited consecutively from one stroke ward in the Department of Neurology, Beijing Tiantan Hospital, Capital Medical University, Beijing, from December 1, 2014 to May 31, 2016.

The inclusion criteria for patients were: aged between 35 to 65 years, with a mild stroke in basal ganglia regions or both in basal ganglia and centrum semiovale, and with no previous history of stroke or TIA. Acute ischemic stroke was diagnosed based on World Health Organization criteria by neurologists^1,25^, and confirmed by brain computed tomography or magnetic resonance imaging. All the participants in this study had a traditional MRI scan. The traditional MRI sequences included T1, T2, DWI (diffusion weighted imaging), FLAIR (fluid-attenuated inversion recovery), MRA (magnetic resonance angiography) and SWI (susceptibility weighted imaging). Stroke patients underwent an MRI as soon as possible once they had been admitted to Beijing Tiantan Hospital. We evaluated the severity of white matter hyperintensities, lacunes, Virchow-Robin spaces and microbleeds on MRI. A separate MRI focusing on high quality DTI was carried out between the 10^th^ to 14^th^ days after their admission when the patients’ conditions were relatively stable. We excluded patients with obvious demyelination on FLAIR. An eligible patient required an informant who knew the patient’s medical history and cognitive status, and who had met with the patient on a weekly basis for at least 5 years prior to recruitment.

The exclusion criteria for patients were: stroke mimics (ie, seizures, migraine), illiteracy, any major physical and mental conditions that may impede cognitive assessments, or acute stroke at cortex or other regions. Major depression defined by a Hamilton Depression Rating Score (HAMD) ≥17, delirium and pre-existing dementia according to their medical history and a score >3.38 on the Informant Questionnaire on Cognitive Decline in the Elderly (IQCODE)^26^ in the 5 years preceding the stroke were also exclusionary. Out of the 56 consecutive patients with mild stroke who were recruited, 23 patients were excluded from our study. The reasons for exclusion were as follows: temporal, brainstem or frontal infarcts (n = 13), contraindications to MRI or dropout during MRI (n = 4), severe hearing impairment (n = 4), and illiteracy (n = 2). The present analyses included 33 mild stroke patients with basal ganglia region infarcts.

### Control subjects

Nineteen healthy control participants from the community were matched with the CI and NCI groups for age, sex, education and handedness. The control participants had no previous history of neurological or psychiatric diseases and no obvious myelination and lacunar infarction according to brain MRI.

This study was approved by the Beijing Tiantan Hospital Ethics Review Board. Informed written consent was obtained from all participants. This study met the guidelines of Capital Medical University, which abides by the Helsinki Declaration on ethical principles for medical research involving human subjects.

## Procedure

### Demographics and clinical profile

Demographic information obtained included age, sex, educational level, handedness, history of hypertension, impaired glucose regulation, hyperlipidemia, hyperhomocysteinemia, peripheral arterial disease, alcohol and cigarette consumption and family history of stroke. The etiological subtypes of ischemic stroke were identified by neurologists as large atherothrombotic infarction (LAA), cardiogenic embolism (CE), small artery occlusion (SAO), undetermined type (UND) and other type (OC) according to the Trial of ORG 10172 in Acute Stroke Treatment (TOAST)^27^. The severity of stroke was evaluated by the National Institute of Health stroke scale (NIHSS)^28^ and mRS^29^, the depression was assessed by HAMD^30^. Basic daily functioning was assessed by the Katz basic activities of daily living (basic ADL) scale^31^, and complex function was assessed by Lawton and Brody instrumental activities of daily living (instrumental ADL) scale^32^. The diameter, locations and circulation systems of lesions were reviewed by two radiologists.

### Neuropsychological assessment

Information about previous cognitive status was collected by a trained neurologist with access to the patients’ medical history and IQCODE scores for 5 years prior to the stroke. The MoCA-Beijing scale was used to screen the global cognitive status of participants^33^. One point was added to the total score for those with education <12 years^34^.

Trained neurologists assessed participants’ cognitive function using a formal battery of neuropsychological tests in line with the NINDS-CSN^35^ neurocognitive harmonization standards. The average assessment time was within 10 days [interquartile range (IQR): 2.00 days] of stroke. The individual tests of the formal battery of neuropsychological test were as follows: (1) Auditory Verbal Learning Test for immediate and delayed verbal memory^36^; (2) Rey-Osterrieth Complex Figure Test (RCFT)-Delayed Recall (CFT-DR) for delayed visual memory^37^; (3) RCFT Copy for visuospatial ability^37^; (4) Animal Fluency Test (AFT)^38^ and Boston Naming Test (BNT, 30-item)^39^ for language; (5) Symbol Digit Modality Test (SDMT) for visuomotor speed^40^; (6) Chinese modified version of the Trail Making Test (TMT)-A^41^, Trail Making Test (TMT)-B^41^, Stroop Color-Word Test-Chinese version (CWT)-Color time^41^ for attention/executive function. The interval between the DTI measure and the administration of neuropsychological assessment was within 12 hours. To allow for the direct comparison of cognitive status in different domains, a Z-score for the neuropsychological tests was calculated. The Z-score was defined as a score that fell within the distribution of scores for norms. The norms used were based on mean scores of each measurement which were derived from a study of healthy-aged community people in China^42^. A total of 339 aged 50–85 years participants were recruited from communities in China with > 6 years of education; 58.7% (199/339) of the participants were female and had no serious physical or neurodegenerative/cognitive diseases. The norms were stratified by age and education. Cognitive impairment was defined as a score of 1.5 standard deviations below the mean on any neuropsychological test of the normative study. According to the Diagnostic and Statistical Manual of Mental Disorders, 4^th^ edition (DSM-4)^43^, finally, we recruited 20 CI patients and 13 NCI patients.

### Image acquisition

Magnetic resonance imaging data were acquired from all participants on a Siemens 3.0 T Prisma MRI scanner (Siemens Healthcare, Erlangen, Germany) at the Functional Neuroimaging Department, Beijing Neurosurgical Institute, Capital Medical University, using a single-shot echo-planar imaging (EPI) sequence (TR = 8000 ms, TE = 60 ms). For each diffusion scan, 30 gradient directions (b = 1000 s/mm²) and a non-diffusion-weighted acquisition (b = 0 s/mm²) were acquired over a FOV of 240 mm × 240 mm²; with a slice thickness of 2 mm and no gap, yielding 2 mm isotropic voxels.

### DTI image preprocessing

Image preprocessing and analyses were performed using PANDA (Pipeline for Analyzing Brain Diffusion Images)^44^, a software package designed for diffusion imaging processing. First, the Digital Imaging and Communications in Medicine (DICOM) files of all subjects were converted into NIfTI images by using the dcm2nii tool embedded in MRIcron. Second, the BET brain extraction was used to delete non-brain tissue from the image, the threshold was 0.25. Third, eddy current distortion and motion effects were corrected by using FSL functions^45^. The diffusion gradient directions were adjusted. A voxel-wise calculation of the tensor matrix and a group of diffusion tensor metrics were then obtained for each subject, including fractional anisotropy maps.

#### Tract-Based Spatial Statistics (TBSS)

Location correspondence was established for subject analyses. To this end, registration of the individual images was applied to a standardized template. The PANDA software then non-linearly registered all of the individual images in their native space to a standardized template in the MNI space^44^. The voxel-wise statistical analysis in TBSS compared group differences only on the white matter skeleton to provide better sensitivity, objectivity and interpretability of analysis for multi-subject DTI studies^46^. The TBSS analysis of FA image was carried out using the FMRIB software library (FSL 4.1.4; http://www.fmrib.ox.ac.uk/fsl).

#### Extract mean diffusion metrics of Atlas-based Tract ROIs

We used the digital white matter atlas JHUICBM-DTI-81 (http://cmrm.med.jhmi.edu/), a probabilistic atlas generated by mapping DTI data of all subjects to a template image. The JHU-white matter atlas was overlaid on the white matter skeleton of each subject in the CBM-DTI-81 space, such that each skeleton voxel could be categorized into one of the major tracts. We then calculated fractional anisotropy at the skeleton voxels within each tract. The regional diffusion metrics (i.e., FA) were calculated by averaging the values within each of 50 regions of the white matter atlas.

### Statistical analysis

Statistical analyses were performed with SPSS Statistics 20.0 (IBM Corporation, New York, USA). Demographic information (age, sex, education, history of hypertension, impaired glucose regulation, hyperlipidemia, hyperhomocysteinemia, peripheral arterial disease, alcohol intake and cigarette smoking habits, family history of stroke) and clinical evaluation (the scores of mRS, HAMD, IQCODE, Instrumental and Basic ADL, the diameter, locations and affected circulation systems of lesions) was compared among the control group, the NCI group and the CI group. Continuous variables, if they were normally distributed, were presented as means ± standard deviations and compared by an ANOVA test. Bonferroni correction was performed in further comparisons between two groups. P value was significant when it was <0.017. Continuous variables, if they were not normally distributed, were presented as median (quartile) and compared by nonparametric test. Discrete variables were compared by Fisher’s exact test.

Voxel-wise TBSS were carried out using a permutation-based inference tool for nonparametric statistical thresholding (“randomize”, part of FSL). In this study, voxel-wise group comparisons were performed using non-parametric, two-sample t-tests in: NCI group versus control group, CI group versus control group, and CI group versus NCI group. The mean FA skeleton was used as a mask (thresholded at a mean FA value of 0.2), and the number of permutations was set to 5000. The significance threshold for between-group differences was set at p < 0.05 [family-wise error (FWE) correction for multiple comparisons] using the threshold-free cluster enhancement (TFCE) option in the “randomize” permutation-testing tool in FSL^46^.

For the atlas-based tract regions, we performed two-sample T tests to compare FA values among three groups for each region of interests (ROI) (p < 0.05 FWE-corrected). There were no significant differences among the control group, NCI and CI groups in age, sex and education; therefore, we did not include age, sex and education as covariates.

Multivariate logistic regression analyses were performed to study the association between FA values in affected regions and impaired cognitive tests in stroke patients.

## Results

### Demographics and clinical profile

Compared to controls, the instrumental ADL scores in the NCI and CI groups were significantly higher (*p* < 0.017). There were no significant differences in age, sex, education, the scores of NIHSS at admission, mRS, HAMD, basal ADL, IQCODE, the prevalence of risk factors, family history and affected circulation system among the three groups. Moreover, there were no remarkable differences in diameter of lesions between the NCI and CI groups.

All subjects were right-handed except for two ambidextrous subjects. The average age of recruited stroke patients (81.8% males) was 56.90 ± 9.23 years (range: 39–65 years) with a mean of 10.56 ± 1.83 years of formal education. The median NIHSS score in stroke patients was 1.00 point [interquartile range (IQR): 2.00 points], and most strokes were classified as SAO (n = 29, 87.88%), with four patients having LAA (12.12%) strokes. The average mRS score was 0.00 point [interquartile range (IQR): 1.00 point] **(**Table 1**)**.

**Table 1:** Demographic information of the control group, NCI group and CI group.

	Control group (n = 19 cases)	NCI group (n = 13 cases)	CI group (n = 20 cases)	p1 value	p2 value	p3 value
Age (years, mean ± SD)	50.74 ± 7.37	54.61 ± 9.11	48.15 ± 8.32	0.40	0.17	0.06
Sex (male/total, %)	13/19 (68.42)	11/13 (84.62)	16/20 (80.00)	0.40	0.48	
Education (years, mean ± SD)	10.68 ± 2.38	9.40 ± 1.73	10.84 ± 1.99	0.84	0.06	0.04
NIHSS at admonition [scores, median (IQR)]	—	2.00 (2.50)	2.00 (2.75)	—	—	0.43
Functional status (Modified Rankin Scale; [scores, median (IQR)])	—	1.00 (2.00)	1.00 (1.00)	—	—	0.37
IQCODE(scores, mean ± SD)	3.07 ± 0.11	3.09 ± 0.06	3.12 ± 0.14	0.58	0.17	0.5
HAMD (scores, mean ± SD)	2.15 ± 2.38	2.14 ± 2.32	2.63 ± 2.41	0.19	0.08	0.86
Right-handedness (cases/total, %)	18/19 (94.74)	12/13 (92.31)	18/20 (90.00)	1.00	0.52	1.00
Instrumental ADL (scores, mean ± SD)	8.00 ± 0.00	8.29 ± 0.73	8.37 ± 0.90	0.17	0.001**	0.014*
Basic ADL (scores, mean ± SD)	6.00 ± 0.00	6.00 ± 0.00	6.07 ± 0.27	0.34	0.99	0.34
**TOAST**						
**LAA (cases/total,%)**	—	**0/13** (**0.00)**	**2/20** (**10.00)**	—	—	**0.51**
**concurrent centrum semiovale infarcts (cases/total, %)**	—	**0/13** (**0.00)**	**1/20** (**5.00)**	—	—	**1.00**
Diameter of lesion (mm, mean ± SD)	—	14.55 ± 4.74	19.02 ± 9.64	—	—	0.09
Left basal ganglia (cases/total, %)	—	10/13 (76.92)	12/20 (60.00)	—	—	0.27
Hypertension (cases/total, %)	13/19 (68.42)	9/13 (69.23)	14/20 (70.00)	1.00	1.00	1.00
Impaired glucose regulation (cases/total, %)	3/19 (15.79)	2/13 (15.38)	6/20 (30.00)	1.00	0.45	0.43
Hyperlipidemia (cases/total, %)	10/19 (52.63)	8/13 (61.54)	16/20 (80.00)	0.73	0.10	0.43
Hyperhomocysteinemia (cases/total, %)	9/19 (47.37)	6/13 (46.15)	6/20 (30.00)	1.00	0.33	0.47
Peripheral arterial disease (cases/total, %)	2/19 (10.53)	3/13 (23.08)	2/20 (10.00)	0.37	1.00	0.36
Current or ever drinking (cases/total, %)	8/19 (42.11)	10/13 (76.92)	14/20 (70.00)	0.08	0.11	1.00
Current or ever smoking (cases/total, %)	8/19 (42.11)	8/13 (61.54)	12/20 (60.00)	0.47	0.34	1.00
Family history of stroke (cases/total, %)	1/19 (5.26)	1/13 (7.69)	2/20 (10.00)	1.00	1.00	1.00
Posterior circulation (case/total, %)	—	4/13 (30.77)	2/20 (10.00)	—	—	0.18

NCI, no cognitive impairment; CI, cognitive impairment; NIHSS, National Institute of Health Stroke Scale; HAMD, Hamilton Depression Rating Score; ADL, Activities of Daily Living Scale; **LAA**, large atherothrombotic infarction**;** *p < 0.017, **p < 0.01. p1: NCI group vs. control group; p2: CI group vs. control group; p3: NCI group vs. CI group.

All patients had acute infarcts in the basal ganglia regions, which included the left caudate nucleus (n = 3), left corona radiate (n = 11); left putamen (n = 1); left internal capsule (n = 10); left thalamus (n = 2); right centrum semiovale (n = 3); right internal capsule (n = 6); right thalamus (n = 3) and right caudate nucleus (n = 4). A total of 33 patients had lesions in the basal ganglia (20 in the left basal ganglia, 12 in the right basal ganglia, 1 with bilateral lesions). There were no significant differences in number of patients with multiple or multi-site infarcts between the NCI group and the CI group.

### Neuropsychological test

The CI group exhibited significantly lower Z-score in every cognitive domain than the control group (*p* < 0.017). Moreover, the CI group demonstrated significantly decreased Z-score in visual delayed memory compared with controls and NCI patients (*p* < 0.017). These data indicated that the CI group had a significantly lower score in visual delayed memory than the control group and the NCI group **(**Table 2**)**.

**Table 2:** Comparison of Z-scores in cognitive function among the control group, NCI group and CI group.

Cognitive function	Controls (19 cases)	NCI group (13 cases)	CI group (20 cases)	p1 value	p2 value	p3 value
**Verbal memory** [scores, median (IQR)]	1.081 (3.053)	−2.674 (4.781)	−3.901 (4.182)	0.472	0.000*	0.394
Total score of AVLT	0.502 (1.881)	−0.500 (2.125)	−1.000 (2.800)	0.250	0.002*	0.668
AVLT-delayed recall	0.001 (2.000)	−1.000 (2.500)	−1.000 (2.000)	0.472	0.046	0.873
**Visual delayed memory** [scores, median (IQR)]	0.171 (1.502)	−0.750 (1.417)	−2.250 (1.625)	0.892	0.000**	0.000**
RCFT−delayed recall	0.171 (1.502)	−0.750 (1.417)	−2.250 (1.625)	0.892	0.000**	0.000**
**Visuospatial ability** [scores, median (IQR)]	0.500 (0.500)	−1.500 (1.000)	−1.750 (4.875)	0.192	0.009**	0.378
RCFT	0.500 (0.500)	−1.500 (1.000)	−1.750 (4.875)	0.192	0.009**	0.378
**Language** [scores, median (IQR)]	2.331 (2.751)	1.167 (4.292)	−1.082 (2.749)	0.991	0.000**	0.117
BNT	1.331 (1.001)	0.667 (2.200)	0.000 (1.333)	0.250	0.116	0.472
AFT	2.003 (1.251)	−0.250 (1.375)	−0.625 (1.688)	0.031	0.000*	0.394
**Visuomotor speed** [scores, median (IQR)]	1.204 (0.503)	−1.100 (1.800)	−1.500 (2.000)	0.000**	0.000**	0.472
SDMT	1.204 (0.503)	−1.100 (1.800)	−1.500 (2.000)	0.000**	0.000**	0.472
**Attention/executive function** [scores, median (IQR)]	4.575 (2.917)	2.117 (6.715)	−1.629 (5.379)	0.472	0.001**	0.394
TMT-A time	1.461 (0.104)	−0.717 (1.232)	−1.292 (2.013)	0.668	0.000**	0.023
TMT-B time	1.232 (0.652)	−0.225 (2.600)	−1.375 (2.517)	0.150	0.001*	0.188
CWT-C time	0.961 (0.252)	−0.333 (1.600)	−0.364 (2.136)	0.061	0.024	0.998
CWT-C correct numbers	0.000 (3.500)	2.500 (1.000)	3.000 (1.375)	0.151	0.029	0.734

AVLT, Auditory Verbal Learning Test; RCFT, Rey-Osterrieth Complex Figure Test; BNT, Boston Naming Test; AFT, Animal Fluency Test; SDMT, Symbol Digit Modalities Test; TMT-A, Chinese modified version of the Trail Making Test; CWT-C, Color-Word Test-Chinese version; *p < 0.017,**p < 0.01. p1: NCI group vs. control group; p2: CI group vs. control group; p3: NCI group vs. CI group.

### Group comparisons of voxel-wise statistics in TBSS

**Figure 1:**
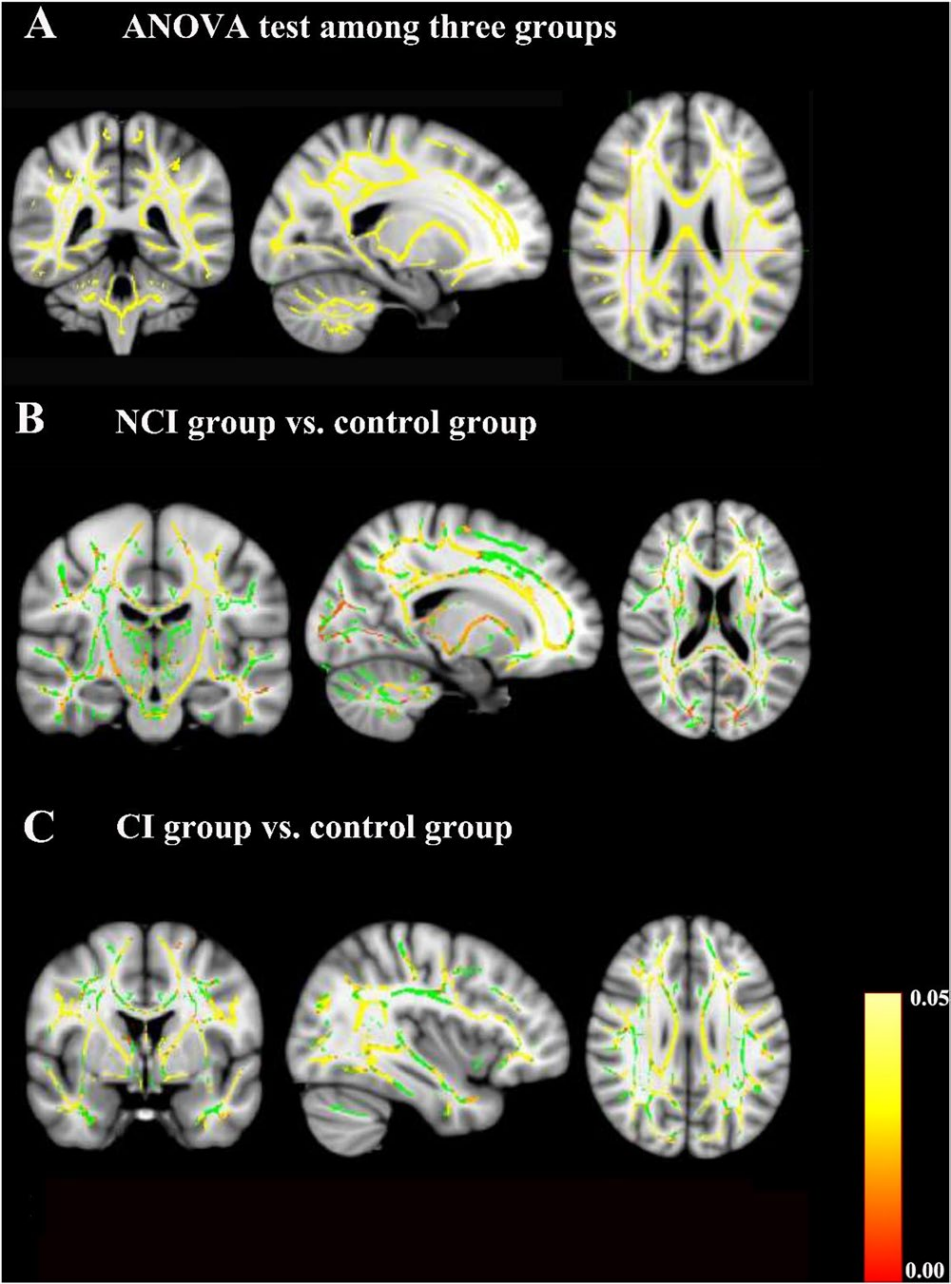
Group comparisons of tract-based spatial statistics (TBSS). Yellow and red indicate WM tracts with significantly decreased FA values. Green indicates WM tracts with no significantly decreased FA values. (**A**) TBSS results among the control group, mild stroke with cognitive impairment (CI) group and mild stroke with no cognitive impairment (NCI) group. The results showed decreased FA values in most brain regions. (**B**) TBSS results between the NCI group and the control group. Decreased FA values were detected in the genu, body and splenium of the corpus callosum, fornix, bilateral corticospinal tract, superior longitudinal fasciculus, inferior fronto-occipital fasciculus, anterior limb of internal capsule, right anterior corona radiate, posterior thalamic radiation, external capsule and left superior corona radiate (*p* < 0.05, FWE-corrected). (**C**) TBSS results between the CI group and the control group. Decreased FA values were visible in the genu, body and splenium of the corpus callosum, fornix, bilateral anterior corona radiate, inferior fronto-occipital fasciculus and superior longitudinal fasciculus, left corticospinal tract, left cerebral peduncle, left posterior limb of internal capsule and right external capsule (*p* < 0.05, FWE-corrected). There was no significant difference in FA values of brain regions between the CI group and the NCI group according to TBSS analysis.

Comparisons of voxel-wise statistics in TBSS among three groups were conducted. Compared to the control group, the CI group showed significantly reduced FA values in genu, body and splenium of the corpus callosum, fornix, bilateral corticospinal tract, cerebral peduncle, bilateral anterior, posterior limb of internal capsule, anterior and superior corona radiate, posterior thalamic radiation, external capsule, superior longitudinal fasciculus, left retrolenticular part of internal capsule, left inferior fronto-occipital fasciculus, left hippocampus, right cingulate gyrus and right posterior corona radiate (Fig. 1 part A, part B and part C). However, when compared to the NCI group, CI group showed no significant difference in FA values.

### Group comparisons of atlas-based tract ROIs

When compared to control group, the CI group showed significantly decreased FA values in several white matter regions the same as in voxel-wise analysis. Furtherly, The CI group showed significantly lower FA values in the right external capsule and right fornix than the NCI group (*p* < 0.05, Bonferroni-corrected).

### Relationship between white matter integrity and neuropsychological function in CI and NCI groups

**Figure 2:**
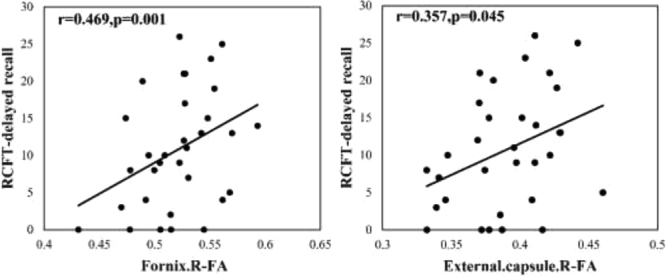
Correlations between the atlas-based regions of interest FA and neuropsychological tests scores in basal ganglia mild stroke patients. RCFT-delayed recall indicates Rey-Osterrieth Complex Figure Test-delayed recall.

Pearson’s correlation analysis showed that FA values in the right external capsule and the right fornix were significantly and positively correlated with RCFT-delayed recall score in stroke patients (Fig. 2). According to a multiple linear regression model, FA value in the right fornix was significantly and positively correlated with RCFT-delayed recall score in stroke patients after adjustments for age, sex, education, diameter of lesions, histories of hyperlipidemia and current or ever drinking (β = 0.002, p < 0.05). In the control group, there were no correlations between RCFT-delayed recall score and FA values in the right external capsule and the right fornix. There was no significantly difference in the presence of white matter hyperintensities, lacune, Virchow-Robin spaces and microbleeds among the control group, NCI group and CI group (Table 3). We also adjusted for the presence of white matter hyperintensities, results were showed that white matter hyperintensities had no effect on FA value in acute mild stroke patients with basal ganglia region infarcts.

**Table 3:** Comparison of important MRI manifestations of cerebral small vessel disease among the control group, NCI group and CI group.

	control group (19 cases)	NCI group v(13 cases)	CI group (20 cases)	p1 value	p2 value	p3 value
**Presence of white matter hyperintensities [cases/total (%)]**	19/19 (100.00)	13/13 (100.00)	17/20 (85.00)	1.00	0.23	0.26
Periventricular white matter hyperintensities [cases/total (%)]	19/19 (100.00)	13/13 (100.00)	17/20 (85.00)	1.00	0.23	0.26
Fazekas scores [scores, median (quartile)]	1.00 (0.00)	2.00 (0.00)	1.00 (0.75)	0.04	0.45	0.02
Deep white matter hyperintensities [cases/total (%)]	5/19 (26.32)	9/13 (69.23)	6/20 (30.00)	0.03	1.00	0.04
Fazekas scores [scores, median (quartile)]	0.00 (1.00)	0.00 (1.00)	1.00 (1.00)	0.87	0.92	0.03
**Presence of lacune [cases/total (%)]**	7/19 (36.84)	9/13 (69.23)	14/20 (70.00)	0.15	0.06	1.00
Basal ganglia [cases/total (%)]	7/19 (36.84)	6/13 (46.15)	11/20 (55.00)	0.72	0.33	0.73
Subcortical regions [cases/total (%)]	3/19 (15.79)	4/13 (30.77)	5/20 (25.00)	0.40	0.70	1.00
Subtentorial regions [cases/total (%)]	0/19 (0.00)	4/13 (30.77)	4/20 (20.00)	0.02	0.11	0.68
Cortex [cases/total (%)]	0/19 (0.00)	1/13 (7.69)	6/20 (30.00)	0.41	0.02	0.20
**Presence of Virchow-Robin spaces [cases/total (%)]**	19/19 (100.00)	13/13 (100.00)	20/20 (100.00)	1.00	1.00	1.00
Basal ganglia [cases/total (%)]	3.00 (3.00)	2.00 (1.00)	2.00 (2.00)	0.73	0.71	0.54
Cortex [cases/total (%)]	4.00 (1.00)	2.00 (3.00)	4.00 (0.50)	0.43	0.15	0.02
**Presence of microbleeds [cases/total (%)]**	0/19 (0.00)	0/13 (0.00)	0/20 (0.00)	1.00	1.00	1.00

p1: NCI group vs. control group; p2: CI group vs. control group; p3: NCI group vs. CI group.

## Discussion

In this study, we investigated the clinical features of cognitive function in acute mild stroke patients with basal ganglia region infarcts. We found that the CI group exhibited prominently lower Z-scores in every cognitive domain than the control group, and that the CI group showed significantly lower Z-scores in visual delayed memory than both the control group and the NCI group. This indicated that visual delayed memory might be the most easily affected cognitive domain in stroke patients with basal ganglia region infarcts. Stroke is known to frequently occur in the basal ganglia, which plays an important role in cognitive function^7^. Previous studies demonstrated that basal ganglia infarcts were associated with memory impairment^8,47,48^. In our study, we further analyzed the memory types and found that basal ganglia region infarcts were related to visual delayed memory rather than verbal delayed memory. This is consistent with our previous study, which showed that visual delayed memory was more likely to be impaired than verbal delayed memory in stroke patients^2^. Memory processing includes encoding, storage and retrieval. Visual and verbal memories have different neural processing mechanisms^49^. A recent study reported that impaired information retrieval was related to subcortical dysfunction, which could result in visual memory dysfunction^50^. This might explain why stroke patients with basal ganglia region infarcts are prone to visual memory dysfunction.

Furthermore, we explored the potential mechanism of CI after mild stroke with basal ganglia region infarcts by measuring white matter integrity with both ROI-based analysis and voxel-wise analysis. Both ROI-based analysis and voxel-based analysis showed that the CI group exhibited significantly reduced FA values across several white matter regions compared with the control group, such as genu, body and splenium of the corpus callosum, fornix and other aforementioned regions (Fig. 1 part A, part B and part C). In ROI-based analysis, data showed that the CI group had significantly decreased FA values in the right external capsule and right fornix compared with the NCI group and the control group. However, in the voxel-based analysis, there was no significant difference in FA values between the CI group and the NCI group. Patients in this study had more left hemisphere BG strokes, however the lower FA values were found in the right external capsule and right fornix in the CI group. As a previous study reported^51^, cerebral ischemia could reduce white matter integrity locally at the primary lesion location due to tissue damage, or remotely as a consequence of anterograde Wallerian degeneration or connectional diaschisis^52^. Global white matter integrity might also play a role in compensatory mechanisms of stroke^53–56^. Structural remodeling and changes in the number of neural pathways in contralesional hemisphere could predict cognitive impairment after stroke^57^. The external capsule and fornix in right cerebral hemisphere did not have fibers directly emanating from the left basal ganglia, however, they were related to cognitive impairment in mild stroke patients with basal ganglia region infarcts, which may be explained by remote effects or connectional diaschisis. This study found reduced white matter integrity in contralesional hemisphere, but not ipsilateral, which supports the functional importance of the contralesional hemisphere. The explanation might be that lower FA in the contralesional hemisphere was due to vascular damage caused by being exposed to the same vascular risk factors that intiated the initial stroke event. Furthermore, based on studies in rats, stroke itself might cause spreading depression in the ipsilesional hemisphere^55^, which might allow the onset of secondary (Wallerian) degeneration of contralesional white matter after stroke.

Given the relatively small sample size of this study, we analyzed the data using two common approaches, i.e. ROI-based and voxel-wise approaches. The ROI methods are typical methods of DTI analysis where specific brain structures are traced and diffusion values are extracted. The ROI methods have the advantage of data sampling from white matter tracts in the native space of the individual, however, this method also suffered from bias. Voxel-wise analysis is characterized by spatial normalization of DTI and statistical analysis include hypothesis test at each voxel and multiple comparison correction. The major challenge for voxel-wise DTI analysis is multiple comparison correction. Therefore, group difference may also be reduced or eliminated during the FA based nonlinear registration procedure^58,59^. In this study, the ROI-based analysis showed that the CI group had significantly lower FA values in the right external capsule and right fornix than the NCI group. However, there was no significant difference in FA value between CI group and NCI group in the voxel-wise analysis. This discrepancy might be explained that voxel-wise analysis had a lower sensitivity for the group difference due to the strict multiple comparison corrections. In additional, the small sample size in this study might also have an impact on the result of voxel-wise analysis, a larger sample size study is needed to verify this result. the two methods might complemented each other in this study.

Multiple linear regression showed that a decreased FA value in the right fornix in stroke patients was related to the RCFT-delayed recall score after adjusting for age, sex, education, diameter of lesions, histories of hyperlipidemia and current or ever drinking (β = 0.002, p < 0.05). However, there were no correlations between FA values and RCFT-delayed recall score in the control group. This meant white matter integrity in the right fornix was not related to visual delayed memory in health controls. Under normal conditions, the fornix was found to be important for episodic memory recall^60^. Previous studies showed that the right fornix was positively associated with memory in non-demented elderly with the APOE ɛ4 allele^61^. It seemed possible that the nonamnestic cognitive impairment was mainly driven by subcortical brain damage disrupting subcortical-frontal connections^62^. We speculated that stroke lesions in the basal ganglia regions could disrupt white matter integrity in the fornix, thereby contributing to the damage of frontal-basal ganglia connectivity and lead to visual memory impairment. A study in ischemic mice found that cognitive deficits were associated with axonal and myelin damage of the external capsule^63^. However, our analysis of multiple linear regression models revealed no association between FA value in the external capsule and RCFT-delayed recall score. This implied that the sample in this study was small and might have led to insignificant findings on the association between white matter lesions in the external capsule and cognitive performance.

Mild stroke patients with basal ganglia regions infarcts exhibited cognitive impairment in all domains compared with controls, including verbal memory, visuospatial ability, visual delayed memory, visuomotor speed, language and attention/executive functions. Mild stroke with basal ganglia region infarcts caused remote white matter damage to areas such as the right external capsule and the fornix. White matter integrity damage in the right fornix may be a potential mechanism for visual delayed memory loss in acute mild stroke patients with basal ganglia region infarcts. The use of DTI proved to be a good tool with which to investigate white matter abnormality and its relationship with cognitive impairment as white matter damage could be an early predictor for cognitive impairment in mild stroke with basal ganglia region infarcts.

This study had some limitations. Firstly, the sample size was relatively small and could have influenced the relationship between decreased FA value in the right external capsule and neuropsychological test score. This association needs to be verified in future studies with a larger sample size. Secondly, in order to reduce the effects of aging on cognition, we recruited patients under 65 years of age, which is below the average age of mild stroke patients as reported in China^64^. Therefore, it is not be possible to extend our findings may not be to all stroke patients. Thirdly, this study was a cross-sectional study with the average assessment of cognition being within 10 days (IQR: 2 days) of stroke. The relationship between WM integrity and cognition was obtained at 2 weeks after stroke. We agree that our findings might not be generalizable to all stroke patients, especially for chronic stroke patients. Fourthly, this study used FA as a neuroimaging index of microstructural white matter integrity like other studies^61,65^, while FA was also sensitive to myelination, axon diameter, fiber organization^65,66^. One study showed that myelin was not an essential component for FA, while intact membranes were the primary determinant of anisotropic water diffusion in neural fibers^67^. Therefore, in this study, the mild stroke patients with basal ganglia region infarcts showed visual delayed memory might most probably be related to white matter integrity damage in the right fornix loss, but can’t eliminate the causes of axonal and myelin damage. Finally, we did not assess the volumes of cortex or hippocampus, which may have an impact on the cognitive function.
